# Staging early Alzheimer's disease progression in Down Syndrome using mixed clinical, biofluid and neuroimaging markers with machine learning

**DOI:** 10.1002/alz70856_100350

**Published:** 2025-12-25

**Authors:** Mina Idris, Fedal Saini, Phoebe Ivain, Leda A Bianchi, Jasmine Wells, Henrik Zetterberg, Peter A Wijeratne, Andre Strydom

**Affiliations:** ^1^ King's College London, London, United Kingdom; ^2^ Institute of Neuroscience and Physiology, Sahlgrenska Academy at the University of Gothenburg, Göteborg, Sweden; ^3^ University of Sussex, Brighton, United Kingdom

## Abstract

**Background:**

Individuals with Down syndrome (DS) are at high risk for Alzheimer's disease (AD), typically developing AD pathology in their thirties. However, the progression of the disease, including the sequence of plasma biomarker, cognitive, and structural brain changes preceding AD in DS, remains unclear. Data‐driven methods, such as the event‐based model (EBM), can estimate the order of changes during disease progression from cross‐sectional data while accounting for baseline variability.

**Method:**

This study applies the EBM to examine clinical, neuroimaging, and plasma biomarker changes in 60 adults with DS and no AD. The aim is to identify the likely sequence of marker changes preceding clinical AD diagnosis and stage individuals along this sequence.

**Results:**

Preliminary analyses suggest that cognitive and plasma biomarker changes in early AD in DS follow a similar pattern to what is observed in sporadic and familial AD. Early changes in plasma amyloid‐beta 42/40 ratio, were followed by memory impairments, neurodegeneration markers (phosphorylated tau 183 and 231, neurofilament light), executive dysfunction, visuomotor deficits, and later neuroinflammation (glial fibrillary acidic protein). Structural MRI will be incorporated alongside plasma biomarkers and cognitive measures to provide a comprehensive multi‐modal disease progression sequence.

**Conclusion:**

This study will refine the timings for cognitive testing, informant ratings, neuroimaging, and blood sampling to enhance understanding of AD progression in DS. The EBM offers individual‐level staging which may enable earlier diagnosis, improve clinical trial design, and optimise biomarker use in this population. Combining modalities promises more insights into preclinical AD stages in DS.

